# Case report: Lateral medullary syndrome with eight-and-a-half syndromes

**DOI:** 10.1097/MD.0000000000034409

**Published:** 2024-02-09

**Authors:** Chun Zuo, Mingmin Zhao, Lei Zhao, Nan Meng, Xing Xing, Na Li

**Affiliations:** a Neurological Intensive Care Unit, Hebei General Hospital, Shijiazhuang, China; b Graduate School of Hebei North University, Zhangjiakou, China; c Hebei Provincial Key Laboratory of Cerebral Networks and Cognitive Disorders, Shijiazhuang, China.

**Keywords:** dysarthria, eight-and-a-half syndromes, oculomotor disorders, Wallenberg syndrome

## Abstract

**Rationale:**

Lateral medullary syndrome is caused by atherosclerosis or embolism of the vertebral artery and its branches or the posterior inferior cerebellar artery (PICA).The eight-and-a-half syndrome is a rare pontocerebellar nerve-ocular syndrome presenting as a one-and-a-half syndrome plus ipsilateral seventh cerebral nerve palsy. The dorsolateral medullary syndrome combined with the eight-and-a-half syndromes is even rarer, so it is important to recognize the features of the classical brainstem syndrome and the eight-and-a-half syndromes.

**Patient concerns:**

Most patients with dorsolateral medullary syndrome combined with eight-and-a-half syndromes have a good prognosis, with recovery occurring within a few weeks to a few months, although a few patients may take longer to recover.

**Diagnosis interventions:**

In the course of disease development, the patient developed dysarthria, dysphagia, hypothermia, ipsilateral Horner sign and ataxia. Computed tomography was performed which showed cerebral infarction in the left brainstem. Cranial diffusion-weighted imaging + magnetic resonance angiography showed acute infarction in the left cerebellar hemisphere, with a high probability of severe stenosis or occlusion in the intracranial and proximal segments of the basilar arteries. This supports the diagnosis of dorsolateral medullary syndrome. The patient’s limited adduction and abduction of the left eye and limited adduction of the right eye, combined with peripheral paralysis of the affected lateral nerve, supported the diagnosis of eight-and-a-half syndromes. The administration of antiplatelet and anti-ester fixation treatment can effectively improve the symptoms and shorten the course of the disease.

**Outcomes:**

After antiplatelet and anti-ester fixation treatment, the symptoms improved and the patient was discharged.

**Lessons:**

Dorsolateral medullary syndrome combined with eight-and-a-half syndromes is a rare clinical condition, and therefore more attention should be paid to the early diagnosis and treatment of such patients.

## 1. Introduction

Lateral medullary syndrome, also known as Wallenberg syndrome, is caused by atherosclerosis or embolism of the vertebral artery and its branches or the posterior inferior cerebellar artery (PICA), or, less commonly, by entrapment of the vertebral artery.^[1]^ Although the occurrence of Horner’s syndrome is well established in patients with dorsolateral medullary syndrome, it is often accompanied by other neuro-ophthalmologic abnormalities.^[2]^ The one-and-a half syndrome, which was first reported and named by Fisher in 1967, is a group of syndromes caused by various causes of pontine lesions with oculomotor disorders as the main manifestation.^[3]^ Wallenberg syndrome with eight-and-a-half syndromes is even rarer. The typical eight-and-a-half syndrome is one-and-a-half syndromes combined with ipsilateral facial nerve palsy, is a rare condition characterized by conjugate horizontal gaze palsy, ipsilateral internuclear ophthalmoplegia (INO), and ipsilateral lower motor neuron facial palsy, caused by injury to the dorsal cap of the pons, involving the paramedian pontine reticular formation (PPRF) and the medial longitudinal fasciculus (MLF), as well as the adjacent facial nucleus and facial nerve. Involved nuclei may include a combination of the ipsilateral seventh and sixth nuclei with the ipsilateral MLF, or a combination of the ipsilateral seventh nucleus and the ipsilateral PPRF with the ipsilateral MLF. The eight-and-a-half syndrome is most often caused by infarcts, multiple sclerosis, and brain stem tuberculoma.^[4]^ This article reports a rare case of dorsolateral medullary syndrome with eight-and-a-half syndromes and provides a retrospective analysis of the relevant antibodies in the context of the literature to improve the understanding of brainstem syndrome superimposition.

## 2. Case presentation

**Figure 1.**
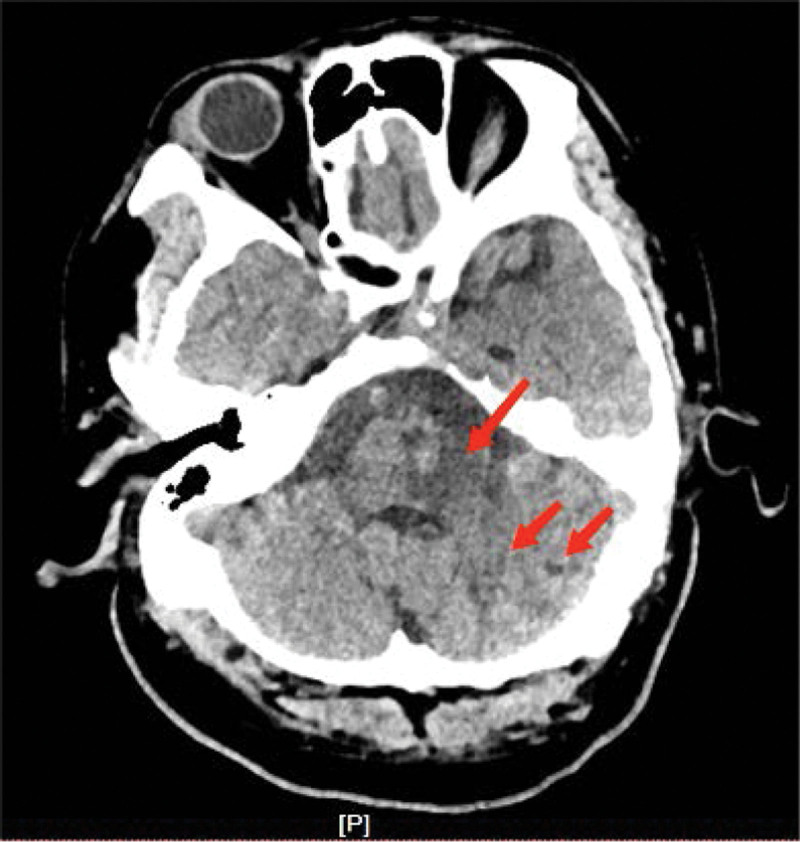
CT: flake low-density shadows were seen in the left cerebellar hemisphere, the left part of the brainstem, and the left pontine arm. CT = computed tomography.

**Figure 2.**
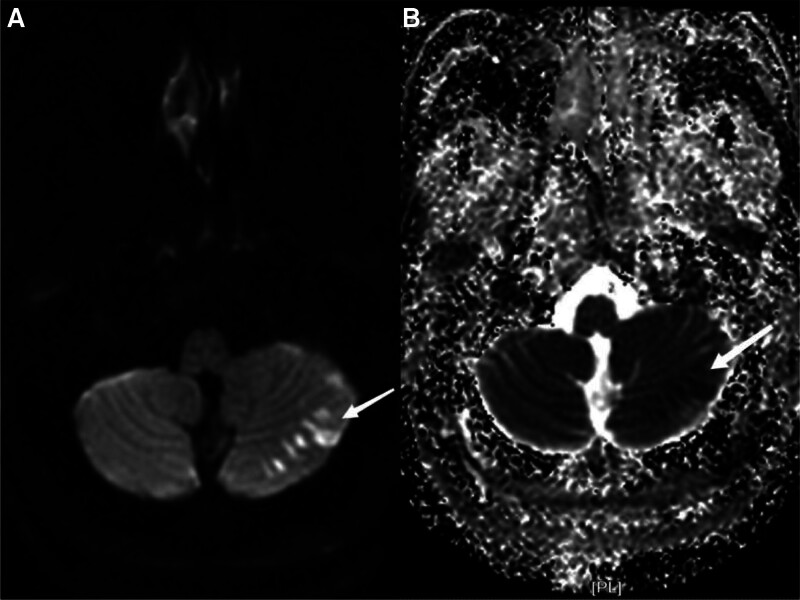
MRI-DWI (A): multiple patchy high signals in the left cerebellar hemisphere; ADC (B): multiple patchy low signals in the left cerebellar hemisphere. ADC = apparent diffusion coefficient, DWI = diffusion-weighted imaging.

**Figure 3.**
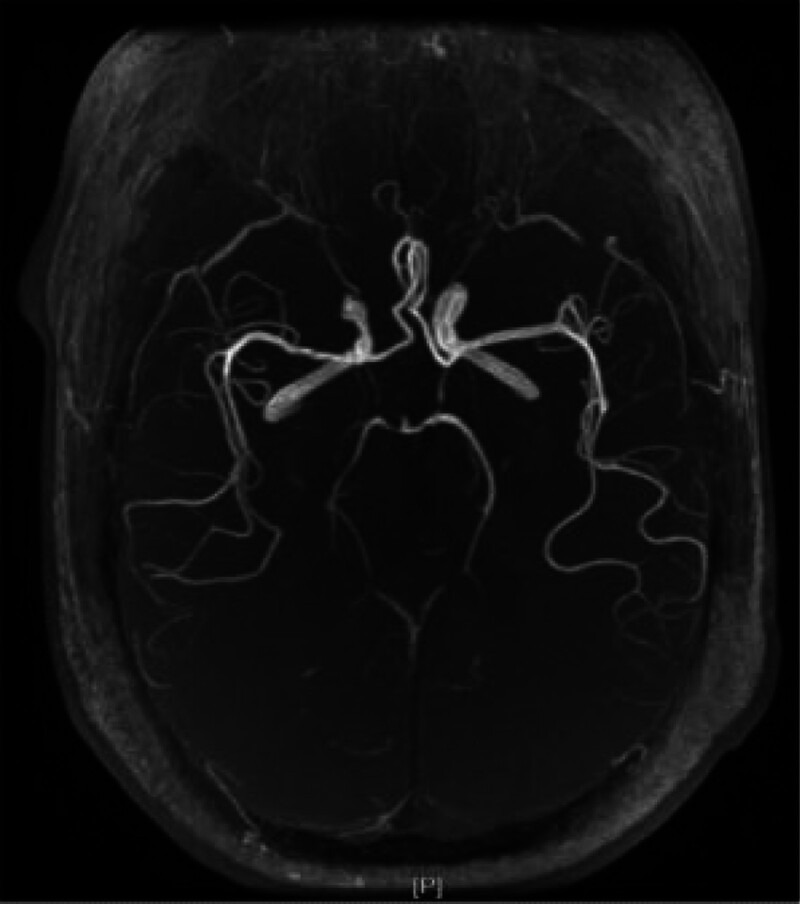
MRA: no clear development in the intracranial segment of bilateral vertebral artery and the proximal segment of basilar artery, but only in the distal segment of basilar artery. MRA = magnetic resonance angiography.

**Figure 4.**
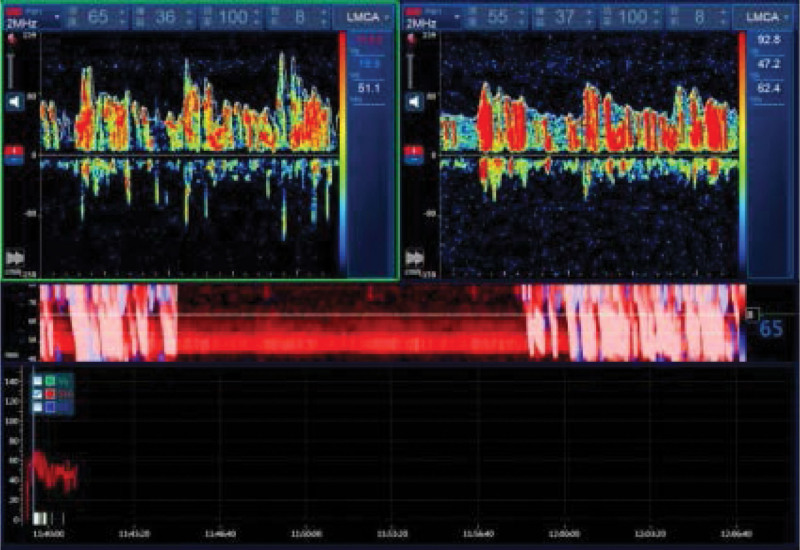
Transcranial Doppler ultrasound foaming test: monitoring single channel and double depth monitoring vessels: left middle cerebral artery resting state: >100 microbubble signals can be seen in about 8 seconds.

The patient, a 47-year-old male, was admitted to the hospital with “Dizziness, unsteadily walking and slurred speech for 3.5 hours.” He has vertigo, dysarthria, choking and coughing with water, difficulty swallowing. Besides, he is conscious, with left pupil diameter of about 2.5 mm and right pupil diameter of about 3 mm, whose sensitive reflex to light. The left eyelid was incompletely closed, the left side of the face was sweatless, the pharyngeal reflex was blunted, the left side of the face and the right side of the limbs had numbness and hypesthesia, the muscle tone of the limbs was normal bilaterally, the muscle strength of the limbs was grade V, and the left ataxia was unstable. On the second day, the patient’s condition progressed with diplopia, limited inversion and abduction of the left eye, horizontal nystagmus visible in the right eye abduction, distorted corners of the mouth, weakness of the left eye closure, shallow frontal lines and nasolabial furrow on the left side, right deviation of the angle of the mouth showing teeth, and right deviation of the sacred tongue. Computed tomography was performed which showed cerebral infarction in the left cerebellar hemisphere, the left brainstem, the left pontine arm and multiple lacunar infarcts in the left frontal lobe, left parietal ventricle and right side of the pontine bridge(Fig. 1). Cranial diffusion-weighted imaging + magnetic resonance angiography showed acute infarction in the left cerebellar hemisphere (Fig. 2), with a high probability of severe stenosis or occlusion in the intracranial and proximal segments of the basilar arteries (Fig. 3). Transcranial Doppler ultrasound foam test showed positive foam test-supporting right-to-left shunt (intrinsic type, massive shunt) (Fig. 4). Blood analysis, C-reactive protein, coagulation function and rapid troponin did not show significant abnormalities. The diagnosis of multiple cerebral infarction was made based on the clinical manifestations and examination findings. After admission, aspirin and atorvastatin were given as a combination therapy, and the patient was discharged after 18 days with improvement of symptoms.

## 3. Discussion

Infarction of the dorsolateral medulla oblongata can cause damage to the trigeminal spinal tract and spinal tract nucleus, spinalthalamic tract, sympathetic inferior fibers, cordura, vestibular nucleus, and nucleus ambiguous. The classic symptoms include ipsilateral facial and contralateral limb pain-temperature sensory disturbances, ipsilateral Horner syndrome and ipsilateral cerebellar ataxia symptoms. The different clinical manifestations of Wallenberg syndrome are influenced by the involvement and severity of the cerebral nuclei and spinal tracts and are therefore dependent on residual perfusion or lateral branch circulation in the region at risk.^[1]^

Dorsolateral medullary syndrome is often referred to as PICA syndrome, but the most common cause of the syndrome is atherosclerotic thrombotic occlusion of the vertebral arteries. In terms of etiology, the TOAST classification is an internationally used classification of cerebrovascular diseases. According to clinical manifestations, neuroimaging and other laboratory tests, the etiology of cerebral infarction can be divided into 5 types: large artery atherosclerotic, cardiogenic embolic, small artery occlusive, other definite etiology and unknown etiology. Hypertension, diabetes, and smoking are common risk factors.^[5]^ The pathogenesis of dorsolateral medullary syndrome is presumed to be large vessel infarction (50%), followed by arterial entrapment (15%), small vessel infarction (13%), and cardiac embolism (5%).^[6]^ This patient is a middle-aged male with many years of previous risk factors for hypertension and diabetes mellitus and transcranial Doppler ultrasound foam test showed positive foam test – supports right-to-left shunt (intrinsic type, massive shunt). Therefore, this patient belongs to the cardiogenic embolic type in very rare cause and has multiple lacunar infarcts with a clear etiology.

**Figure 5.**
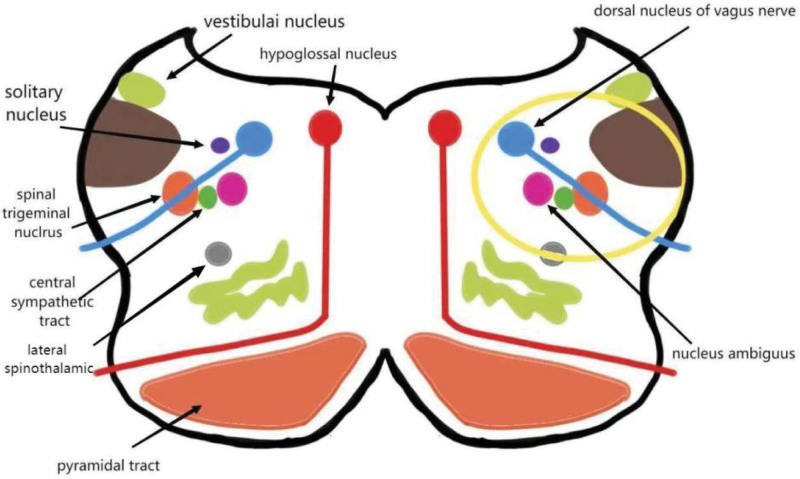
Lesions causing dorsolateral bulbar syndrome (yellow area).

This patient has the basic features of the Wallenberg syndrome (Fig. 5): little sweating on the left side of the face and pupil narrowing are localized in the left sympathetic inferior fibers.^[5]^ Left-sided facial sensory disorder is localized in the left nucleus of spinal trigeminal tract.^[7]^ Hyperalgesia of the right limb is located in the left spinalthalamic tract; dysarthria, dysphagia, weak lift of the soft palate, and sluggish gag reflex are located in the nucleus ambiguus.^[1]^ Dizziness and nystagmus are localized in the vestibular nucleus.^[7]^ The left eye superior vision restriction was localized in the left nucleus of the oculomotor nerve and its descending fibers; the left eye abduction restriction is localized in the left nucleus of the abducens nerve and its descending fibers; the left peripheral facial palsy is localized in the left nucleus of the facial nerve and its descending fibers; and the synthesis is localized in the left medulla oblongata and the left pons and midbrain. The dorsolateral medulla syndrome is the most common form of all posterior circulation ischemic infarcts in clinical practice, and MRI + diffusion-weighted imaging is the gold standard for confirming this diagnosis. Clinically, different individuals present with different symptoms. The dorsolateral medullary syndrome may also present with uncommon clinical symptoms such as hiccup, tongue deviation to the contralateral side of the lesion, and ipsilateral hypotonia of the arm.^[6]^ It is believed that the diagnosis of Wallenberg syndrome requires the following 2 items: a lesion in the medulla oblongata – dysarthria or dysphagia; a lesion in the dorsolateral medulla oblongata – dysesthesia, Horner’s sign, ataxia. According to the diagnostic criteria, this patient had dysarticulation and dysphagia, accompanied by ipsilateral facial, contralateral hemisensory disorders, ipsilateral Horner’s sign, and ipsilateral ataxia, therefore could be diagnosed in combination with cranial MRI.^[8]^

**Figure 6.**
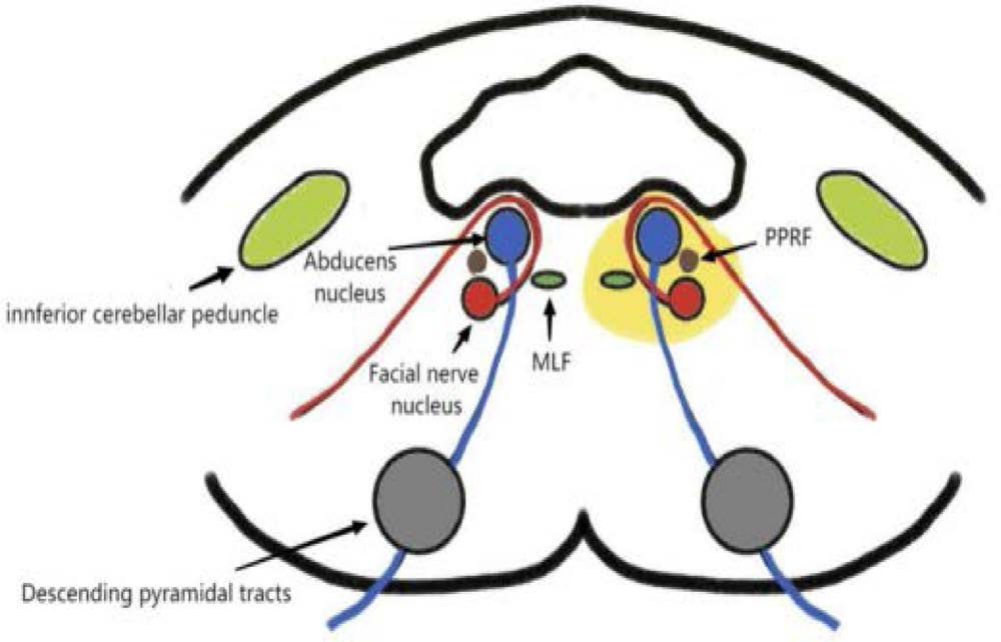
Anatomic sites of lesions causing eight and a half syndromes (yellow areas).

The eight-and-a-half syndrome, as a special and uncommon type of the dorsolateral medullary syndrome combined with oculomotor abnormalities, was first described by Eggenberger in 1998.^[9]^ It refers to coupled horizontal gaze paralysis and INO plus ipsilateral seventh cranial nerve paralysis.^[10]^ It is a rare pontine neuro-ophthalmic syndrome.^[9]^ Dorsolateral medullary syndrome combined with eight-and-a-half syndromes is much rarer and, therefore, its recognition has considerable diagnostic value.^[11]^ Conjugate horizontal gaze is a binocular movement that synchronizes the eyes in one direction of gaze, and these motor controls originate in the brain and brainstem. The main horizontal gaze control center is located in the PPRF which sends signals to its ipsilateral abducens nerve and the contralateral medial longitudinal bundle, and lesions in this region can lead to horizontal conjugate gaze paralysis. INO is a condition in which the affected eye has limited internal ophthalmoplegia and ataxic horizontal nystagmus occurs in the contralateral eye during abduction. INO originates from a lesion in the MLF, a pair of crossed axonal tracts located near the brainstem, which receives signals from the contralateral PPRF and then sends them to the ipsilateral oculomotor to coordinate conjugate eye movements. Lesions that cause INO include stroke, occupying lesions, and demyelination. The lesions affect both the PPRF and MLF on the same side, resulting in one-and-a-half syndrome. One-and-a-half syndrome, first described by Fisher, is a condition consisting of ipsilateral conjugate horizontal gaze palsy (“one”) and ipsilateral internuclear eye muscle palsy (“half”).^[12]^ One-and-a-half syndrome are most often caused by multiple sclerosis, infarction, hemorrhage, trauma, basilar artery aneurysms, brainstem arteriovenous malformations, and tumors.^[11]^.The only remaining eye movement is contralateral eye abduction.^[13]^ In rare cases, the lesion may affect the PPRF, MLF, and the ipsilateral lateral nerve bundle as it surrounds the abducens nucleus in the pontine tegmentum. This lesion may cause ipsilateral peripheral facial palsy, called the eight-and-a-half syndrome.^[9]^ Some patients also have other clinical manifestations.^[14]^ Thus, identification of this syndrome allows precise localization of the lesion to the ipsilateral side of the subpontine tegmentum.^[11]^ This patient had limited internal and abduction of the left eye and limited internal retraction of the right eye, combined with peripheral paresis of the affected lateral nerve. Based on his clinical features and neuroimaging, eight-and-a-half syndromes were considered (Fig. 6).^[4]^

The patient started with dorsolateral medullary syndrome and gradually progressed to eight-and-a-half syndrome. The reason for this is the proximity of the 2 anatomical structures and the vascular localization. It is generally believed that PICA originates from the ipsilateral vertebral artery, or partially from the basilar artery, and the bulbar branch of PICA supplies the dorsolateral part of the bulbar, while the parapontine perforator of the anterior inferior cerebellar or basilar arteries supplies the tegmentum of the pontine. Therefore, this embolism involves multiple deep perforating branches of the basilar artery. In addition, combined with the positive foaming test-supporting right-to-left shunt (intrinsic type, massive shunt), the presence of unclosed foramen ovale, and risk factors such as obesity and sleep apnea, this patient was considered to have a cardioembolism resulting in embolization of multiple penetrating branches of the vertebrobasilar artery in different spaces and at different times, thus presenting a superposition of the more rare brainstem syndrome with a higher disability rate and the patient’s quality of life should be affected, so early diagnosis and treatment, control of risk factors, and prevention of complications should be carried out in clinical practice.

The outcome of eight-and-a-half syndromes or their variants and eight-and-a-half-plus syndromes is usually good, with recovery in a few weeks to a few months, especially with improved oculomotor deficits.^[4]^ However, in a few cases there is no significant improvement in oculomotor deficits even after more than 6 months, which may require longer time to recover.^[15]^ The overall prognosis of Wallenberg syndrome is also better than most other acute ischemic infarcts.^[6]^

In addition to the classic dorsolateral medullary syndrome and the eight-and-a-half syndromes, we should also pay attention to the superimposed brainstem syndrome and its variants which are easy to miss due to the need for detailed cranial nerve examination, such as oculomotor examination.^[4,16]^ Therefore, it is important to recognize the features of the classical brainstem syndrome and the eight-and-a- half syndromes in order to localize the lesion and determine the appropriate treatment plan.^[17]^ This case highlights the importance of bedside examination and the ability to correlate clinical findings with known brainstem Neuroanatomy to help select the examination.^[18]^

## Acknowledgments

The authors would like to thank Na Li for her assistance in writing this manuscript.

## Author contributions

**Formal analysis:** Mingmin Zhao, Lei Zhao.

**Methodology:** Xing Xing, Chun Zuo.

**Supervision:** Na Li.

**Resources:** Nan Meng.

**Conceptualization:** Nan Meng, Xing Xing.

**Writing – original draft:** Chun Zuo.

**Writing – review & editing:** Mingmin Zhao.
